# Why can't we make research grant allocation systems more consistent? A personal opinion

**DOI:** 10.1002/ece3.4855

**Published:** 2019-02-10

**Authors:** Roger Cousens

**Affiliations:** ^1^ School of BioSciences The University of Melbourne Parkville Victoria Australia

**Keywords:** agency, allocation, assessors, impact, innovation, merit, ranking, research grant, scoring

## Abstract

Uncertainty is expected to enter into our grant allocation processes at many points, not limited to those directly involving assessment by peers. The selection of grants for funding is thus prodigiously low in statistical power and will remain so. The replacement of current systems with some form of lottery, as has been proposed, seriously risks weakening the quality of applications. Opportunities exist for agencies to encourage and reward greater clarity and innovation in research outcomes.

## Abstract 2

Recent papers have shown that peer review of grant applications is little better than random selection. Attention here is drawn to additional sources of uncertainty and the inherent low statistical power of our adjudication systems. Suggestions are made for improved focus on outcomes and the encouragement and reward of novel proposals.

## 1 THE PARADOX: HIGH RESEARCH QUALITY BUT LOW POWER IN JUDGING RELATIVE MERIT

Research grants are the lifeblood of most ecologists and evolutionary biologists. Without grants most of us can do little research, we cannot support students, we cannot publish as many papers, our reputations will not progress—and our positions may even be terminated! With so much on the line, it is no wonder that the time of the year when grants are announced is highly stressful. We know that the odds are often stacked against us, with perhaps less than a 20% success rate in some schemes. Even so, given the amount of effort that goes into a full submission it is still disappointing to receive a rejection. If the reviewers find flaws, weaknesses or poor explanation, we can accept these because they were our fault and because we can correct many of them next time around. But it is the inconsistencies and unpredictabilities that frustrate us, combined with the lack of opportunity in most systems to mount any defense. The extreme cases readily come to mind: the glowing praises accompanied by poor ratings (though we are happy to see the converse!); the occasional reviewers who appear not to have understood our arguments or who have opposing entrenched views on the same topic; or the time when an application just missed out one year, only for an improved version to be ranked low down the next!

As I will discuss, there are many unavoidable reasons why error, in a statistical sense, enters into our funding allocation systems. The great shame is that in a highly competitive process just a single mediocre review—it does not even have to be a bad review—can thwart a promising line of enquiry. And the frustration is not just on the applicant's side. Even if asked to assess an application in my stated “area of expertise,” there is such a wide range of research activity that I cannot be completely even‐handed. I believe that I am a good judge of quality, but my knowledge base is bound to be incomplete and uneven. The same will be true for a committee: even if aided by peer reviews, a committee will come across some applications outside the detailed knowledge of the entire panel. But we do our job and rank every application as best we can.

Grant allocations are highly affected by chance. This conclusion is not at all new and is hardly surprising. Peer review will always have low (statistical) power: it is founded on very low replication (few reviewers per proposal) and a very high variance (value judgements by people with a wide variety of knowledge and views: also see Boudreau, Guinan, Lakhani, & Riedl, 2016). Formal studies of the consistency of the allocation of scores or ranks have found that it is not possible to discriminate reliably between a large proportion of grants (Boudreau et al., 2016; Graves, Barnett, & Clarke, 2011), especially if the standard of the applications is high. Indeed, allocation decisions may be little or no better than random (Avin, 2018; Pier et al., 2018) even if peer review is accompanied by additional mechanisms such as review committees (Graves et al., 2011). It has thus been argued that we waste large sums of money in assembling and judging information related to our selection criteria, and that research funding systems would be far cheaper and no less effective, if based on a modified lottery rather than on peer review (Fang, Bowen, & Casadevall, 2016; Guthrie, Ghiga, & Wooding, 2017; MacKay, Kenna, Low, & Parker, 2017). As persuasive as the negative analyses of peer review are, our “best” researchers whose applications regularly appear in the upper percentiles are unlikely to support a completely random allocation. We would still need some form of quality control to ensure that only “worthy” projects—however we choose to define this—go into the lottery and that clearly weak projects do not (Fang & Casadevall, 2016). And, surely, few of us would argue against the philosophical concept that our ideas should be open to scrutiny?

Funding agencies try extremely hard to make their systems as fair as possible, continually adjusting them to reduce opportunities for bias, specifying selection criteria with increasing clarity and occasionally introducing excellent innovations. Likewise, large numbers of volunteer assessors and committee members work hard to give fair and objective assessments. It remains, however, that scientists whose reputations are based on their ability to test the veracity of their ideas and results with a high level of confidence are judged by funding allocation systems with inherently low power. They will mistrust the system even if there is a high level of transparency (Abdoul et al., 2012).

I would not be considered the foremost authority on research funding systems, but I have considerable experience as a participant in funding schemes. I completed my PhD over 35 years, 150 + papers and 3 books ago and for 20 years I have been a full Professor in one of Australia's top research Universities Although I have had many failures in my 50 + career research applications, I have received numerous grants from both basic and applied funding agencies; I am asked to review several grant applications each year, both as a formal assessor and as a mentor to colleagues; and I have served on grant allocation committees of various forms. Much of my experience is based on the Australian and British systems, but I have had extensive discussions with North American colleagues. I have tried to draw on generic issues rather than specific agencies. I have accessed the copious detail provided online by funding agencies and the extensive international literature on review processes. What I present, however, are highly subjective, personal conclusions that may find resonance with my peers and may, perhaps, turn out to be useful. Few of them are original, but a number are seldom expressed in print. All relate to the inherent, and mostly insoluble, low discriminatory power of our selection systems. I first consider the many factors contributing to the variation in the scores of assessors and then suggest some adjustments that could at least reduce frustrations for funding scheme participants. I do not suggest any ways in which to make our systems perfect!

## 2 SO MANY REASONS FOR DECISIONS TO BE UNPREDICTABLE!

The basic assumption of our allocation systems is that the relative merits of applications can be determined by a selection of experts provided with a research proposal and other background data. The data include a plethora of information which the particular agency considers to reflect on merit. Some examples of these are as follows: details of research inputs, outputs, and other achievements of the applicants; the status of their institutions and their research facilities; the feasibility of the project; research costs; ethical issues; various scientific, social and other benefits if the project succeeds; communication plans; training; and involvement of under‐represented groups. Merit is very difficult to define, and its measurement can be highly contentious in any field (e.g., Williamson, Colley, Foley, & Cooper, 2018). Funding agencies do their best to ensure that their methods are transparent and stand up to scrutiny. Most commonly, they express merit in a semiquantitative way, resulting in a score that allows applications to be ranked. In very general terms, the statistical model for this is.

Merit Score=Merit Predictors+Error

In practice, the task is not to produce an accurate ranking of proposals, but categorization: to distinguish the top *x* % from the bottom (100‐*x*) %.

The detail of the merit model varies between agencies and schemes, depending on their aims and—to a considerable extent—their historical development. Each aspect of the model can potentially affect the level of uncertainty:

### 2.1 The objectives of the funding scheme

This determines what information is relevant to the determination of merit. The more specific the objectives of the scheme and the clearer the instructions, the more likely it is that the applicant will provide what the agency is seeking. Lack of a clear agency vision, or merely poor communication of that vision, will mean a wider range of expectations by applicants, assessors, and committees. Frequent alterations in vision and instructions to applicants may add to uncertainty, while variation in objectives between funding schemes may make it harder for applicants to “hit the mark” in any given one: an example might be where a basic scientist applies for applied research funds.

Clearly, each funding agency needs to have its own aims and will change these as it sees fit, so this source of uncertainty will remain. We should encourage clarity by agencies in documentation and their adoption of additional communication measures, such as “roadshows,” and the use of portfolio managers whose role is to liaise with research providers. Employers of researchers need to mentor their staff to identify with the agency, rather than just seeing it as a funding source; if there are portfolio managers, a working relationship needs to be developed with them.

### 2.2 The data required from the applicant

This constitutes the formal basis on which the application's merit is judged. The information required can range from a very concise statement of the proposed research to an extremely comprehensive document addressing numerous issues in great detail, or a combination of these (e.g., Mervis, 2016). The more complex the task, the greater the opportunity for uncertainties to be introduced.

Some data requirements may be unambiguous and can be achieved precisely, such as a list of publications and grants received in the last 5 years. But for most criteria that is not possible. Even the best way to describe the science of the intended project can be unclear: how much detail to give in a limited number of pages, what knowledge can be assumed of the assessors? I have had some assessors criticize the lack of detail on replication and randomization of field plots, whereas others have clearly been content to read about the underlying logic of the treatments. Other requirements may be extremely vague, such as “broader impacts” (Nadkarni & Stasch, 2012; Watts, George, & Levey, 2015), resulting in uncertainty for the applicant and thus variance in their response. For example, the Australian Research Council Discovery Project (ARCDP) 2018 scheme asked applicants for a 100 word *Benefits and Impacts Statement* and provided a table in a FAQ sheet of 25 things that might be considered for inclusion. The difficulty of such tasks is exacerbated by the fact that applicants usually do not know what an assessor or committee would regard as exemplary: ARCDP gives an example for a *Benefits and Impacts* assessment for a good project: “an important application with an innovative approach that should engender new public policies leading to improved outcomes. It is also value for money*.*” but gives no advice on how to judge innovation, improved outcomes, or value for money.

Many research provider organizations now supply excellent interpretations of funding agency expectations, liaising closely with them and providing guidelines and training to researchers. They also draw upon their own researchers who are past members of granting committees. While such actions may assist in achieving better ranks for their organizations' projects, comments from colleagues at other institutions suggest that the quality varies considerably.

### 2.3 The selection of assessors

The implicit assumption of many funding schemes seems to be that assessors will recognize merit when they see it, using their experience and their own standards. If the sample size was to be large or variance small, then differences among assessors would average out and we could be comfortable with the result. But that is far from the case. Sample size is extremely small (seldom more than five peer reviewers and often as few as two) and the variance is extremely large, such that there may be almost no correlation between assessors (Avin, 2018; Pier et al., 2018). Some applications will therefore be unduly affected by outlier assessments that may be unsound. If we were designing our own research project, such low statistical power would be unacceptable—to ourselves or to a journal! Our ability to increase the number of assessors, however, is highly constrained, by the burden that we place on volunteer reviewers and the load on administrators. If we were to calculate the number of assessors required to raise the power of our judgements to an acceptable standard, we would no doubt find the solution impossible to resource.

Many agencies understand the issue of small numbers of peer reviewers and have developed mechanisms through which they identify and adjudicate on specific cases of error, usually at a committee stage. A few agencies even seek out instances of unfair reviews by allowing applicants to comment on their reviewers' assessments. In some systems, there is a second tier of peers or “expert” panellists (or merely subsets of committee members) whose rankings of a larger sample may be combined in some way with scores from the first tier. Indeed, a two‐tier informal assessment system of this kind could be replaced by a more formal method of calibration of the external reviewers (MacKay et al., 2017), though this seems to be uncommon. The second tier increases the number of replicate assessors and allows us to remove some sources of variance, but there will be less opportunity for a close one‐on‐one match of assessors to applications in this second tier. Adjudication at the committee stage is constrained by the limited diversity of expertise of its members, with decisions likely to be subject to the range of interindividual dynamics common in negotiations (e.g., Zubin & Brown, 1975).

So, can we decrease assessor variance? No matter how objective an assessor tries to be, the nature of their task is to conduct a subjective assessment of qualitative information; most of the information provided to them is qualitative. Assessors vary in a great many ways, all of which can be expected to affect their scores. For example, they vary in their philosophical views of the discipline, their pre‐established biases and opinions (such as prior views on particular types or topics of research, or of particular researchers), their standards of expectation, their response to risky innovation versus solid traditional studies, their tendency to award high versus low scores, the time and care taken for each assessment, their experience, their fallibility (some will notice errors more readily than others—see Baxt, Waeckerle, Berlin, & Callaham, 1998), their readiness to accept the opinions of others (Park, Peacey, & Munafò, 2014), the seriousness they place on certain assessment criteria, their stance on the relative merits of applied versus basic research, and even their tolerance of poor writing.

Standardization of assessors could be attempted through some form of training, though further impinging on their time. However, a study of peer review of journal submissions found a slight and only transient impact from assessor training (Schroter et al., 2004). Information posted on web sites or sent directly to assessors is a way to provide the opportunity for self‐education, if assessors choose to take it, but it is unclear the extent to which the information is actually consulted. Many of us are no doubt prone to a degree of self‐confidence: we have assessed grants so many times, surely by now we understand our role? *Et mea culpa*: while I may regularly reacquaint myself with funding system‐specific procedures, I seldom take the time to study all the advisory documents and links provided each year by each funding scheme.

Assessment variance will also be affected by the way that assessors are allocated to applications. It is usual to attempt to ensure that at least some assessors are knowledgeable on the topic of the application. Administrators may use names of people who are cited in the application, or they may match project keywords with researcher areas of interest on some database. Formal algorithms are available for this, which can remove some of the subjectivity (Mimno & McCallum, 2007). In a very diverse field, however, the level of knowledge match will vary and some applications may well be assessed by people with very limited relevance to the research topic. Indeed, there may actually be a deliberate process of assigning pairs of reviewers to applications, one close and one further from the topic. It has been found that assessors tend to give lower scores to applications in their own areas of research and to those projects which are more innovative (Boudreau et al., 2016; Gallo, Sullivan, & Glisson, 2016). If the funding scheme seeks assessors internationally, some of them may have no experience with the funding agency or its expectations, and this is likely to be another source of variation in scoring. The use of double‐blind assessment rather than full disclosure can overcome some sources of bias (Budden et al., 2007), but within narrow fields of research and small research communities the improvements may be small since the identity of applicants may be obvious.

### 2.4 The measurement system

Assessors are usually asked to award either a numeric score (a percentage) or to allocate the application to a sequence of grades (such as A to E). Academics are notorious among their students for their variation in marking of essays: some are easier to impress than others, while there may be reluctance to use the very top or bottom grades. Use of a formal rubric can help with marking consistency (Reddy & Andrade, 2010) and most agencies now provide assessors with some form of marking guidelines. However, it is still a highly subjective decision whether an application is in the “top 10% of applications,” or some other band. Gamesmanship by some assessors may also play a part in the variance of scores. In a system in which only a small proportion of applications succeed, many assessors will be aware that unless they award a mark in the top band of the scale the project has little chance of being funded. That is also how they believe other assessors will behave. So, their decision becomes a binary one: top band if it should be funded, a lower band if not (it is of little consequence which).

### 2.5 The classification of applications prior to assessment

The applicant may be asked to assign the application to a particular category, thus ensuring adjudication by an appropriate discipline committee or so that it is ranked alongside others within a similar target group, such as stage of career, ethnicity, or gender. This can have a considerable effect on the score likely to be awarded. An animal ecology project, for example, is likely to be judged very differently by committees whose remits are specified as, say, biology, zoology, environmental science, or ecology; the committee members can have quite contrasting areas of expertise and expectations, and the competing projects or researchers may have quite different characteristics.

Interdisciplinary applications continue to be a particular problem (Adams, Loach, & Szomszor, 2016). Despite being recognized widely as under‐represented in research funding and likely to lead to novel scientific advances, they are not handled well by most funding agencies. Although some agencies have a separate scheme or committee for interdisciplinary projects, others allocate interdisciplinary applications to one discipline panel with a request that there should be discussions with other discipline panels. As a result, interdisciplinary research applications have a lower success rate than single‐discipline applications (Bromham, Dinnage, & Hua, 2016). A review of procedures for such projects is seriously needed by many agencies.

### 2.6 The weighting of components of merit

There are a great many criteria that funding agencies seek to have considered in an assessment of the merit of an application. Some of these are scored as individual criteria, while others are aggregated. In many schemes, all scores are weighted equally (Abdoul et al., 2012); in others, the weights can be unequal or the weighting can be up to the assessor (Langfeldt & Scordato, 2015) and not necessarily reported. To receive a high overall score, all components need to be rated highly; the greater the number of categories, the less influence any particular one of them will have. The astute assessor will be aware of this when they are considering their marks.

Although as many as six criteria may require separate scoring (Abdoul et al., 2012), the two most prominent criteria are subjective assessments of the research team and of the quality of the proposed research. A considerable effort is demanded of the researchers to present extensive factual data, mostly their past outputs, citation data, impact factors of the journals, grant successes and awards. The logic here is that researchers with the best track records will continue to produce the best work (but see Smaldino & McElreath, 2018). Thus, it is quite common to receive assessor comments based on quantitative aspects of track record, such as the number of publications being lower than it “should” be, bearing in mind all sorts of caveats. However, even the most explicit of metrics, such as researcher output or citation rates, are highly questionable as measures of quality or innovation and their use elicits behavior that further erodes their utility (Smaldino & McElreath, 2018).

The relative importance of the perceived strength of the research team and the quality of the project have received considerable discussion: should support go to the project or the person (Germain, 2015)? Agencies may sometimes make awards directly to highly achieving researchers without the need for them to provide full project details. However, the two criteria are usually given similar weight in grant scoring systems. Thus, no matter how original and exciting the research proposal, its score will be pulled down if it is submitted by a research team without a consistently strong track record.

Scores are also given for various other criteria including benefit, outcomes, communication, and institutional arrangements. If each additional criterion is scored separately, then the influence of researcher and project scores on the overall assessment will be reduced (unless this is counteracted by increasing their weight). Many agencies, however, ask assessors to give a single grade to a composite set of these minor merit criteria (Nadkarni & Stasch, 2012; Watts et al., 2015). The mere act of amalgamation will introduce an inadvertent weighting to each criterion: if several criteria are combined in one assessment category, such as “Broader Impacts”, each one will have less influence on the total and the combined score is more likely to be mediocre. Indeed, if a criterion is “buried” in a miscellaneous category, most assessors will consequently interpret it as being unimportant and will give it less attention than others.

Individual assessors will differ in which of the component criteria are most strongly represented in their score. [In fact, the same issue arises in judgement of researcher track record and quality of the project: how much more important is number of publications than, say, grants received; how much more important is the research question, the detailed description of each experiment and its overall feasibility?] Assessors also vary in the confidence with which they feel they can assess the minor criteria. When I am asked to judge communication plans, benefits to society and attention to under‐represented minorities, I feel completely adrift, with little guidance from the agency to help me. How do I decide objectively on a score for things in which I have little background or interest?

### 2.7 The procedures of the committee

A committee of the funding agency commonly has a part in the assessment process. They have procedures for adjusting scores or ranks to solve individual problems when they are alerted to them; however, they do not review every application in equal measure, so many errors in the system may still go un‐noticed. Committees may also have a role in “balancing” the research portfolio (e.g., Baerwald, 2013), perhaps moving projects between “funded” and “unfunded” to reduce or increase the proportion of the budget on certain topics, or to achieve additional criteria and quotas—including social, equity, or political directions. Although committees no doubt follow protocols for these procedures, to applicants it would seem to add further uncertainty in how their project will be treated.

## 3 SOME THINGS WE COULD CHANGE

By presenting a “warts and all” review of the many issues leading to high levels of uncertainty, my primary aim has been to help inexperienced researchers to better appreciate the multiplicity of reasons why the assessments of their proposals are so highly unpredictable. With demand so much exceeding availability of funds, we must accept that even our best proposals will often not be funded and, galling though it might be, less impressive projects—in our own estimation—will be funded. Indeed, it could be argued that it is pointless tinkering with our peer review systems: if a large proportion of good proposals will miss out anyway because funds are seriously limited, does it matter that a few good proposals miss out because of a fallible system for judging merit? Eventually, the best projects will find funding: it may just take a while.

Over time, our agencies have identified flaws in their systems and have made effective changes. This process will need to continue. But, given that the lead time for changes can be considerable, are there things that can be identified now and that we can start to examine? There is little chance that there will ever be the increased investment in resources necessary to achieve an order‐of‐magnitude increase in statistical power, but there may be ways to achieve modest improvements. The research literature on peer review and funding decisions has been very active in recent years and makes fascinating reading.

### 3.1 Why not a lottery?

Given our inability to discriminate among proposals of high merit (Pier et al., 2018), several authors have suggested that a modified lottery system would be cheaper (e.g., Fang & Casadevall, 2016) and perhaps more honest that what we have now. If a system is, in essence, no better than a lottery, why not formally make it one? In my view, research funding agencies are unlikely to move to a lottery system, whatever the cost savings. Government and industry providers motivated by free market economics and politics are unlikely to favor a system where their money is allocated primarily by chance, even if it does cost less to administer. There will also be a conservatism against radical change, due to the considerable effort invested into the improvement of our current review systems over many decades. One concern that I find highly convincing is that a lottery would result in less thorough development of project ideas by applicants. As researchers, it is also a matter of pride that we would want our quality and credentials to be judged by our peers.

### 3.2 Better reward for originality?

One completely unintentional outcome is that our systems apparently tend to suppress innovation (Guthrie et al., 2017)—the very thing that researchers, fund providers, and managers all agree is a crucial component of the development of knowledge. The agencies charged with distributing funds frequently express their desire for projects to *break new ground*; their lexicon commonly includes *innovation*, *significant advances,* and *transformative ideas*. Our current adjudication systems, however, focus on criteria that are either immediate or backward‐looking: for example, researcher past success, elegance of hypotheses, soundness of design, and technical feasibility of the current proposal. Future consequences, if included at all, are not given a high weighting or are subsumed within broader “impact” criteria where they have little influence. Several studies have found that speculation, innovation, and impact are less likely to be rewarded by basic science funding schemes than the rigor of the scholarship (Abdoul et al., 2012; Boudreau et al., 2016; Braben, 2004; Guthrie et al., 2017). Proposals that are highly speculative (thus scoring low on a *likelihood of success* criterion) and submitted by relatively inexperienced researchers (scoring low in terms of *track record*) may well be the ones needed to pave the way for future advances but tend to receive low scores from peer review (Boudreau, Guinan, Lakhani, & Riedl, 2012). So, researchers playing the funding game may tend to play safe (Kaplan & Vakili, 2015). It is not the fact that we are devoid of new ideas: we need to encourage them in grant applications and reward them accordingly.

One approach is to set aside funds specifically for more speculative projects (Spier, 2002). For example, in the USA the National Institutes of Health (NIH) now has a High‐Risk, High‐Reward Research Program including Pioneer Awards, New Innovator Awards, and Transformative Research Awards, where applications need to be: “transformative, catalytic, synergistic, cross‐cutting, or unique” (NIH, 2015). As with more conventional funding programs, there are serious issues of developing appropriate criteria and assessment methods (Bammer, 2016): how do we define innovation; how do we recognize it when we see it; who are the most appropriate people to judge it? Innovation, to my mind, is inextricably linked to the issue of impact, because the greater the innovation the more likely it is to have a wide‐reaching impact. Rather than agencies inventing completely new funding schemes, an alternative might be to have some sort of “flag” that is raised within current schemes, when an assessor recognizes something highly original. Perhaps a box to tick; then, if the proposal does not make the funding cut‐off, the committee can look carefully to see whether it should be moved in the ranking. Merely asking an assessor to give yet another pseudoquantitative score, in this case for originality, would have little influence once it is averaged with other criteria.

It is sometimes argued that major innovations are more likely to come from young researchers and that preferential funding should be given to them (there are also workforce‐planning reasons for giving preference to the young: Daniels, 2015). Granting agencies now often have separate application categories for early career researchers, increasing their chances of success, and sometimes special awards for those who have demonstrated aptitude for originality (Gewin, 2012). The argument that young researchers are the best innovators is partly based on well‐known studies of the ages at which science Nobel laureates tend to do their ground‐breaking work (Jones & Weinberg, 2011; Simonton, 1988) and a selective list of innovative (IT) companies formed by young entrepreneurs (Levitt & Levitt, 2017). It is often overlooked that the age–Nobel relationship has become less marked since c.1980, with a mean age of breakthrough now being 45–50 years of age (was this change due to the maturity of knowledge or an age‐bias in our funding system: Levitt & Levitt, 2017?). Not so young!

### 3.3 Increase the importance of impact?

Funding agencies want their research to have an impact or benefit, whether this be measured on social, economic, utility, or scientific scales. Yet they usually give impact a low effective weight or merge it with other criteria and, consequently, the “impacts” section of a grant is treated by many researchers as just an afterthought in a sales pitch. The applicant is left to decide which impacts they should discuss, with little guidance about what the agency truly values. Believing that governments are increasingly keen to see economic benefits to their nation, even from basic research, it is common for applicants to try hard to impress with economic statements based on the value of some industry. As an assessor (and one who has published papers using simple economics—Cousens, Doyle, Cussans, & Wilson, 1986), I always feel highly uncomfortable trying to judge the persuasiveness of this crystal ball‐gazing and seldom find the arguments convincing.

If an agency is seriously interested in economic outcomes, then researchers should expect to provide proper economic modelling within their proposal. If agencies want to see innovative basic science, then researchers should provide cogent arguments about the longer‐term advancement of knowledge beyond the project, detailing specific challenges and current impediments to progress. Little is achieved for the agency by scientists stabbing around in the dark trying to identify weak or speculative economic, environmental, or social implications; the time of applicants is wasted, and assessors have little idea how to judge this aspect of merit.

An allocation system rigorously based on judgement of outcomes would also require a philosophical change by many research agencies. If any assessment criterion is to be taken seriously, then it must be weighted highly in the scoring system relative to other criteria. Researchers would then need to be provided with much greater clarity; with so much at stake, applicants and assessors cannot be left to read between the lines. It would also require a motivational change by many researchers. In applied research, it is usual to start with what needs to be *achieved*, and only then do we design the appropriate research pathway to get there. This is in distinct contrast to much fundamental research which starts from what the researcher desires to *find out*. This is more than mere semantics. If the project is funded, where will it lead us; what will logically come next —depending, of course, on the results? A focus on achievement of goals—albeit still in terms of understanding—calls for the raising of research horizons, to consideration of where we are heading rather than where we are now. It requires much more from researchers than the production of vague statements that “the project will lead to increased understanding of x.” We expect to be judged, by promotion and appointment committees, on our research trajectory and the outcomes that we are seeking, so why not in a grant application?

Alternatively, applicants might be asked simply to tick a box to indicate which of several impacts they would like their proposal to be considered, with perhaps 100 words to explain why that impact applies to their project. Whether or not this short statement is persuasive might be judged by the administering committee who share a common set of expectations, rather than the peer reviewers. As a scientific assessor, I would much prefer to be asked to judge the merit of a project in terms of the development of science rather than vague nonscience.

### 3.4 Increase the clarity of expectations?

In reviewing the many causes of inconsistency in the scoring of research proposals, it becomes apparent that the most pervasive problems are not simple variability in the data provided by the applicants or the opinions of the assessors. Uncertainty arises in many ways in the subjective assessment of merit, for example: the vagueness of expressions in language and their interpretation (linguistic uncertainty: Carey & Burgman, 2008; Wallsten, Budescu, & Erev, 1988) by funders, applicants, and assessors alike; the ability of both funding agencies and applicants to express their true intent; the incompleteness of knowledge of—or provided to—the assessor (epistemological uncertainty: Walker et al., 2003). These can be reduced to some extent if there is an awareness of their existence (Carey & Burgman, 2008). Guidance by funding agencies tends to focus on rules and procedures rather than intent. Online instructions and guidelines for applicants and for assessors are largely ineffective as a means of communication or training. Research support teams in applicants' organizations can be effective in liaising with the funding agencies in an interpretative and feedback role, but the lead really needs to come from the funding agencies.

If agencies regard particular types of research as having high merit, they need to communicate this effectively: do they want the best edited proposals, proposals with the best spark of originality, or proposals that are likely to achieve particular aims (no doubt they want all three, but in my experience, assessors vary in their preferences and thus their scoring)? Many agencies seem to want a wide range of everything and reserve their judgement until they see what is on offer. For example, an announcement that “research on climate change is a priority” is not very helpful. Does the agency want to advance basic understanding; to identify how to achieve climate change mitigation; or to assist managers or legislators in making decisions? Do they really want *any* climate change research; how will they then objectively judge which are the most deserving of funds? I wonder if the problem is that many agencies see themselves as peer review sieves rather than bodies setting research directions: is that seen instead as the purview of the government or the “research community”?

Agencies can have a valuable role in the initiation of research proposals more directly. Discipline‐based conferences and symposia organized by societies and research journals are highly *in*effective ways of achieving discourse on research directions: they are designed primarily for one‐way delivery of research results to a passive audience (Cousens, 2017). Research funding agencies could sponsor various forms of workshop, differing in format and outputs. These include conventional mini‐conferences based on communication between researchers, from which collaborations and funding applications might emerge; “sandpits” and “ideas labs” of invited participants with guaranteed funding of their resulting best proposals (though these can be highly contentious—e.g., Robertson, 2013); and workshops designed specially to elicit dialogue, leading to papers that pose challenges to fellow researchers (Cousens, 2017). Although research funding is seriously limited, and researchers may baulk at the idea of some of this being siphoned off for discussion rather than action, any appreciable improvement in the rate and direction of knowledge advancement should surely be encouraged?

## 4 DO WE NEED TO REINVENT OUR GRANT ALLOCATION SYSTEMS?

Although our grant allocation systems have evolved over decades, we have by no means attained perfection. There has been considerable effort into ensuring that changes are made to address problems as they become apparent and to try new ideas that appear logical. To some extent, there has thus been a degree of adaptation. But, like evolution in nature, it is possible that not all changes will have been adaptive: there may have been the equivalent of fixation by drift and the survival of deleterious alleles (e.g., Travis et al., 2007). The funding environment and the constraints placed upon us have been changing in a highly directional manner. Is it therefore possible that our funding systems will, at some point, reach an evolutionary dead‐end and more radical adjustments will become necessary; this is the essence of the argument of those proposing a modified lottery.

Have we reached that point? Philosophically, I do not like the idea of a lottery. And no other alternatives to our current systems are under active consideration. Some countries once had a system of direct grants to all faculty members, but competitive systems are here to stay. If we abandon one highly imperfect system, it is unrealistic to expect any replacement system to be unaffected by the same issues. It is inherent in a system of judging relative merit that it will be plagued with the problems of subjectivity, epistemological and linguistic uncertainty, and high variance. In a steadily changing funding environment, we must anticipate the need for ongoing change. We should be inviting suggestions for change, from users of the systems, administrators and those who have made research funding a subject of research, and evaluating the best options.

## CONFLICT OF INTEREST

The author declares no conflict of interest.

## AUTHOR CONTRIBUTIONS

RDC wrote the manuscript in its entirety.

## Data Availability

There are no data associated with this paper.
